# A longitudinal qualitative study on physician experience in managing multimorbidity across the COVID-19 pandemic in Odisha, India

**DOI:** 10.1038/s41598-024-60473-0

**Published:** 2024-06-04

**Authors:** Pranab Mahapatra, Krushna Chandra Sahoo, Sanghamitra Pati

**Affiliations:** 1 Department of Psychiatry, Kalinga Institute of Medical Sciences, KIIT University, Bhubaneswar, Odisha India; 2 Department of Health Research, Health Technology Assessment in India (HTAIn), Ministry of Health and Family Welfare, New Delhi, 110001 India; 3 ICMR-Regional Medical Research Centre Bhubaneswar, Chandrasekharpur, Bhubaneswar, Odisha 751023 India

**Keywords:** COVID-19, Chronic care, Physician’s management, Multimorbidity, Longitudinal qualitative, India, Health policy, Health services, Public health

## Abstract

While many studies have documented adverse impact of multiple chronic conditions or multimorbidity on COVID-19 outcomes in patients, there is scarcity of report on how physicians managed these patients. We investigated the experiences and challenges of clinicians in managing patients with multimorbidity throughout the COVID-19 pandemic in Odisha state, India. To understand the factors influencing illness management and the adaptive responses of physicians alongside the evolving pandemic, we followed a longitudinal qualitative study design. Twenty-three physicians comprising general practitioners, specialists, and intensivists, were telephonically interviewed in-depth. Saldana’s longitudinal qualitative data analysis method was employed for data analysis. COVID-19 pandemic initially diverted the attention of health systems, resulting in reduced care. With time, the physicians overcame fear, anxiety, and feelings of vulnerability to COVID-19 and started prioritising patients with multimorbidity for treatment and vaccination. All physicians recommended teleconsultation and digital health records to benefit chronic illness care during future public health crises. The findings underscore the transformative potential of physician resilience and adaptation during the COVID-19 pandemic, emphasizing the importance of prioritizing patients with multimorbidity, incorporating teleconsultation, and implementing digital health records in healthcare systems to enhance chronic illness care and preparedness for future public health crises.

## Introduction

Chronic diseases are the leading cause of global deaths every year. Low- and middle-income countries (LMICs) are disproportionately affected, accounting for over three-quarters of deaths from noncommunicable diseases (NCD) globally^1^. There has been a steady rise in the NCD burden in India, the most populous LMC nation^2,3^. At the same time, there has also been a concomitant rise in multimorbidity, defined as the presence of two or more chronic conditions in an individual, which is a growing concern in healthcare, especially among ageing populations. According to studies, one in four people in India has at least one chronic condition, and 8.9% have multimorbidity. For those attending primary healthcare facilities, more than half of patients (54.7%) had chronic illnesses, of which 28.3% had multimorbidity^4,5^.

Chronic illnesses and multimorbidity present several challenges for individuals and health systems. Affected patients need to undergo long periods of treatment, for which they have frequent outpatient visits and hospital stays at different points of care. These measures increase the household costs associated with health care through a high out-of-pocket expenditure (OOPE) in the form of spending on medicine, medical tests and transport, which increases with each additional chronic physical or psychiatric condition^4–6^. Concurrently, the rising number of persons with chronic illnesses causes strains on health systems and care delivery through increased healthcare use, resource strain, and compromised healthcare quality^7^. LMICs, traditionally oriented towards managing acute health conditions and chronic infectious diseases, often have weaknesses in health systems for chronic illness care^7,8^. These challenges in chronic illness care get magnified during public health disasters^9,10^.

The COVID-19 pandemic has significantly impacted healthcare systems worldwide. The rapid influx of patients requiring hospitalisation and intensive care overwhelmed healthcare systems and engaged most healthcare workers, disrupting non-COVID-19 healthcare services like outpatient visits, diagnostics, and managing chronic illnesses or their complications^11–14^. Social distancing measures, lockdowns, and fear of contracting the virus have decreased access to healthcare facilities. These delays in timely and appropriate care have resulted in poorer health outcomes and increased mental health issues^15,16^. Furthermore, individuals with chronic illnesses and multimorbidity are at a higher risk for severe outcomes from COVID-19, including hospitalisation and death. With their multilayered vulnerability, the care and management of these patients became more critical during the pandemic, underscoring the need to address them at all times^17^.

Chronic illness care was affected in India as the pandemic continued for an extended period with surges of three distinct waves^18^. Though the state of Odisha in eastern India has faced similar disruptions in health services during cyclones and floods^19^, the pandemic affected these services for a more extended period. Our previous studies found that patients with a chronic illness or multimorbidity had difficulties with medical consultations, diagnostic services, daycare procedures, and emergency services^15,16,20^. However, our studies also revealed that two third of the patients with a single chronic illness and more than a third of those with multimorbidity maintained continuity in their treatment and doctor consultations^15,16^. Many patients managed their chronic illness by contacting their primary physician, a nearby private clinic, pharmacists or informal health providers. Some others replaced their routine consultations with teleconsultations and subsequent self-care measures^16,20^. As patients overcame their challenges to seek treatment, physicians also responded to deliver chronic illness care.

Recognizing the critical need to prioritize patients with multimorbidity, who face heightened vulnerability during public health crises, holds significant importance. While existing studies have predominantly focused on patient experiences, there remains limited evidence regarding how healthcare providers across diverse settings responded to the challenges posed by the COVID-19 pandemic. The demonstrated resilience and adaptation of physicians during this crisis underscore their transformative potential in understanding and shaping healthcare systems. Moreover, insights gained from the positive impact of these adaptive strategies are invaluable for informing healthcare policies and practices. This knowledge contributes to the cultivation of resilience in the face of unforeseen challenges, paving the way for more effective and patient-centred responses to complex health scenarios during future emergencies. Understanding healthcare providers' experiences becomes instrumental in developing robust healthcare systems capable of addressing a spectrum of public health disasters, including pandemics, floods, cyclones, and devising strategies to cope with the escalating burden of chronic illness. In this context, the present study explored how physicians adapted their practice across the three waves of the COVID-19 pandemic in Odisha to ensure continuity of care in patients with chronic illness and multimorbidity.

## Methods

### Study design, settings, and participants recruitment

We undertook a qualitative study in Odisha, India, during the COVID-19 pandemic to explore physicians' personal experiences while managing chronic illness and multiple long-term conditions, their challenges, and how they delivered care. General practitioners, specialists in Internal Medicine, and physicians of other specialities who managed patients with chronic illnesses in their usual practice settings were invited to participate in the study. Most of these physicians had managed COVID-19 patients, while a few were afflicted with the virus themselves, needing treatment with isolation, with one participant passing away due to COVID-19. These participants represented a variety of specialities, practice settings, and experience levels.

To ensure the inclusion of physicians from various specialties, we used a purposive selection method with a maximum variation sampling approach. These healthcare professionals were chosen from a variety of step-down care facilities, including primary, secondary, and tertiary settings, to provide a comprehensive representation of Odisha's national healthcare network. The selection process included clinicians from the public and private sectors, as well as from rural and urban areas. General physicians were chosen from primary healthcare centres in both rural and urban areas, while specialists were drawn primarily from urban tertiary healthcare facilities, with one specialist representing a district hospital. All the participants voluntarily participated. PM, the first author, is a clinician (psychiatrist) based at a private medical college hospital in the capital city, while SP, another author, serves as the director of the National Medical Research Centre situated in the capital city of the state. All authors actively participated in managing the COVID-19 pandemic within the state. The recruitment of participants was facilitated through the authors' personal and institutional networks. Their average age was fifty, seven participants were women, and they all had an average of twenty-four years of experience. The details of the physicians interviewed are provided in Table 1.

**Table 1:** Details of physicians interviewed.

Physician ID	Interview dates			Speciality	Sex	Years of Experience	Institution and work context
	First wave (June 2020)	Second wave (April 2021)	Third wave (January 2022)				
P1	+	+	+	General physician	Male	8	Rural primary health centre
P2	+	×	×	Ophthalmologist	Female	30	Urban private hospital
P3	+	+	×	Intensivist	Female	21	Tertiary hospital in a city
P4	+	+	+	Psychiatrist	Male	31	Tertiary hospital in a city
P5	+	+	+	Community medicine	Male	32	Health Administrator in Government
P6	+	+	+	Pulmonary medicine	Male	41	Urban private clinic
P7	+	+	×	Pulmonary medicine	Female	12	Tertiary hospital in a city
P8	+	+	+	Intensivist	Female	22	Tertiary hospital in a city
P9^a^	+	×	×	Nephrologist	Male	33	Tertiary hospital in a city
P10	+	×	+	Psychiatrist	Male	13	District hospital in a city
P11	+	+	+	General physician	Female	25	Urban primary health centre
P12	+	+	×	Hematologist	Male	32	Tertiary hospital in a city
P13	+	+	+	Gastroenterologist	Male	27	Tertiary hospital in a city
P14	+	+	+	Oncologist	Male	26	Tertiary hospital in a city
P15	+	×	+	Endocrinologist	Male	34	Urban private hospital
P16	+	×	+	Otorhinolaryngologist	Male	22	Government hospital in suburban area
P17	+	+	×	Nephrologist	Male	8	Urban private hospital
P18	+	+	+	Oncologist	Male	23	Urban private hospital
P19	+	+	+	Cardiologist	Male	25	Urban private hospital
P20	+	+	×	Ophthalmologist	Male	26	Urban private hospital
P21	+	+	+	Rheumatologist	Male	22	Tertiary hospital in a city
P22	+	+	+	Internal medicine	Male	22	Tertiary hospital in a city
P23	+	+	+	Internal medicine	Female	24	Rural primary health centre

+ indicates participated in interview and × indicate did not participate in the interview.

^a^Passed away due to COVID-19.

The first series of semi-structured interviews were conducted in June 2020, coinciding with the first COVID-19 wave, when healthcare services were most strained. We contacted twenty-three physicians, and all agreed to participate in the first series of interviews conducted over the telephone at their convenience. As the pandemic continued, two more surges in the number of cases, the second and third waves, were experienced. These waves led to a return of all COVID-19-related precautionary measures, like personal protection measures and movement restrictions affecting healthcare services. In this context, the research team decided opportunistically to extend the initial study by reaching out to the participating physicians for interviews in the second wave (August 2021) and the third wave (January 2022). Eighteen physicians were available for interview in the second wave and sixteen in the third wave. The subsequent interviews examined how their clinical practice and healthcare settings evolved to address chronic illness care. The study adopted a longitudinal qualitative design in line with the approach of Saldana^21^. The timeline of events related to COVID-19 in Odisha is presented in Table 2.

**Table 2:** Timeline of events related to COVID-19 in Odisha.

Dates	Events
March 2020	RTPCR test started at RMRC Bhubaneswar
March 4, 2020	A State crisis management committee was formed to make policy decisions regarding cluster containment
March 13, 2020	COVOD-19 pandemic is declared a state disaster. The Department of Health and Family Welfare ordered the closure of cinema halls, swimming pools, gyms, and educational institutions except for holding examinations until March 31
March 15, 2020	A student who returned from Italy and belonged to Bhubaneswar tested positive for COVID-19—the first reported case in Odisha
March 16, 2020	The Government issued an order for travellers returning from foreign countries to register themselves and remain in home quarantine for 14 days
March 18, 2020	The Government issued The Odisha COVID-19 Regulations, 2020. These regulations require government and private hospitals to have dedicated COVID-19 isolation facilities. Later, after a few days, nurses and other paramedics were recruited, thus increasing the health workforce in the state
March 21, 2020	The Government announced a lockdown in five revenue districts and eight towns in the state until March 29. The lockdown involved (i) suspension of public transport services, (ii) closure of all commercial establishments, offices, and factories (iii) banning the congregation of more than seven people at any public place
March 24, 2020	With two cases in the state, the Government extended the lockdown to the entire state till March 29. Establishments engaged in the supply of essential goods and services were excluded from this lockdown
March 25, 2020	Nation-wide lockdown enforced by the central Government between March 25 and April 14, extended till May 3
March 26, 2020	Started the process of setting up 1000-bed COVID-19 hospitals in two Bhubaneswar-based medical colleges
March 28, 2020	The Government ordered District collectors and Municipal Commissioners to use closed schools and hostel buildings as temporary shelters for migrants
April 3, 2020	The Government added the following provisions to the Odisha COVID-19 regulations 2020: (i) additional duties and responsibilities of hospitals and local bodies, such as infection control measures in hospitals, among others. (ii) state government or empowered officers can declare any government or private hospital as a COVID-19 hospital
April 3, 2020	The first containment zone was declared in a Bhubaneswar locality to prevent community transmission from patient No. 5
April 7, 2020	The Epidemic Diseases (Amendment) Ordinance, 2020, promulgated. This Act was concerned with preventing the spread of dangerous epidemic diseases
April 7, 2020	Wearing masks was made compulsory for the people stepping out of their houses and were included in the regulations
April 22, 2020	The Government announced specific measures to support the personnel fighting COVID-19 in the state, like protection and support for death during work
April 22, 2020	Registration and 14-day quarantine for migrant workers returning to the state
June 1, 2020	Shutdown over weekends announced
June 1, 2020	Door-to-door surveillance of ILI/ SARI and comorbidities (DM, Hypertension, TB) was initiated, and people with symptoms and comorbid patients were tested for COVID-19 by RT-PCR
July 2020	Rapid antigen test started in Odisha
July 15, 2020:	Plasma banks were established, and convalescent plasma therapy started
January 1, 2021	Cinema halls and theatres were allowed to open at 50 percent capacity. Social, religious, entertainment, sports, academic, marriage, and cultural functions were allowed with a maximum gathering of 200 people following the COVID-19 guidelines
January 8, 2021	Classroom teaching for Class IX and XI began
January 16, 2021	COVID-19 vaccination started first for health workers. Later, for frontline workers, elderly persons, and people above 45 years with comorbidities
January 26, 2021	First wave- COVID-19-positive cases increased in July, peaked in mid-September (daily caseload crossed 4000), and dropped off in January (daily caseload below 100)
April 2021	The second wave started
Jan 2022	The third wave of the COVID-19 pandemic

### Data collection and management

**Figure 1:**
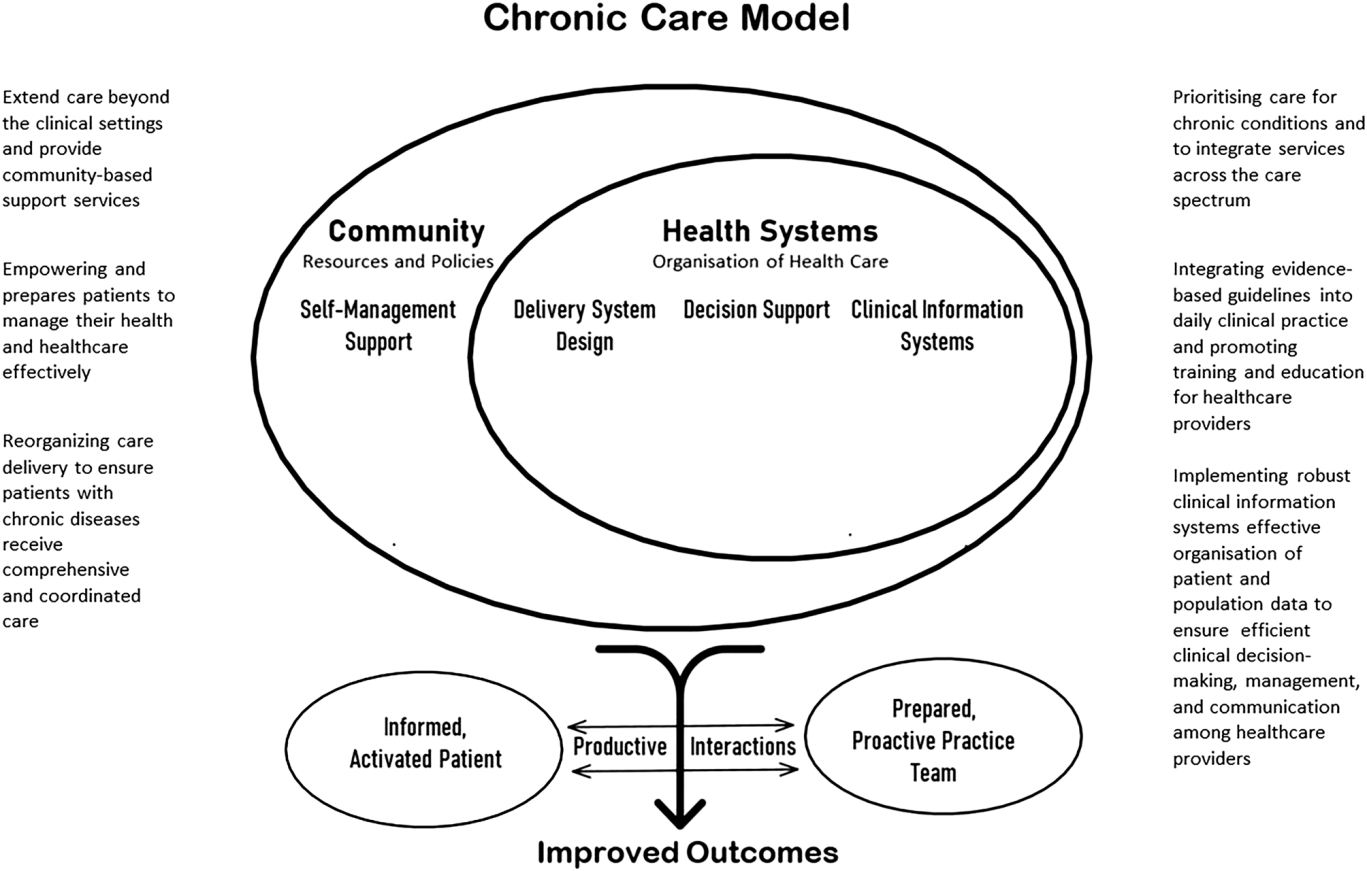
The chronic care model adopted from Wagner, 1998.

In-depth interviews were conducted over the telephone, scheduled at the convenience of the participants. These interviews were semi-structured and followed an interview guide (Table 3) prepared earlier in regional language in this case ‘Odia ‘. The interview guide was developed by integrating principles from the Chronic Care Model (CCM) (Fig. 1) and incorporating considerations from the prevailing COVID-19 protocols during each wave. The CCM framework served as a foundational structure, focusing on key elements such as healthcare organization, community resources, self-management support, delivery system design, decision support, and clinical information systems. In tandem, the interview questions were tailored to explore how chronic care practices and healthcare delivery were impacted by the evolving COVID-19 protocols during each wave. This approach aimed to capture insights into the challenges and adaptations within chronic care management, considering the dynamic context shaped by the pandemic.

**Table 3:** Interview guide—semistructured questions asked to the physicians.

1. Request and note the physicians’ experience and place of work	
2. How were patients with chronic illnesses and multimorbidity managed before the COVID-19 Pandemic? Routine and emergency physician consultations, Interventions like daycare, chemotherapy, and physiotherapy	
3. What were their apprehensions towards the COVID-19 Pandemic? What precautions did they take?	
4. How did their practice change due to the COVID-19 Pandemic?	
5. How did the pandemic affect their healthcare setting and patient care? Explore their routine consultations, emergency services, and services like chemotherapy, physiotherapy, and daily health activities	
6. How did they improvise and adapt to address chronic illnesses care– the consultations, allied services, interventions and investigations, and supplying medications?	
7. What were the challenges faced by their patients? How could they assist in mitigating these? What were the innovative approaches they adopted?	
8. Explore what changed between the interviews—their practice approach, healthcare settings, government initiatives and scientific knowledge and guidelines	
9. Did the overall approach to chronic illness and multimorbidity care change during this period?	
10. Suggestions to improve the healthcare system to address situations similar to the COVID-19 pandemic?

All interviews were conducted by the authors, PM, a practising psychiatrist having experience in qualitative research, and SP, a public health expert in multimorbidity research with a medical background, and have command on local language (Odia). The interviews extended from 15 to 45 min and were digitally recorded. All the interviews were trascribed and translated into English language. Data were anonymised and stored in a password-protected computer folder. To ensure consistency and accuracy in qualitative interview transcripts, a rigorous process involving standardized interview protocols, comprehensive training for interviewers, regular debriefing sessions, and cross-checking of transcripts against audio recordings was implemented.

### Data analysis

Transcripts from the first series of physician interviews were analysed and thematically coded with Miles and Huberman's approach^22^. The transcripts were coded using the MAXQDA-2022 software^23^. The preliminary codes were grouped using pattern coding and further organised into categories and themes. The codings were carried out initially by PM and KCS in successive steps, they were continuously discussed with SP. Themes and categories were reassessed until a consensus was reached on the final coding structure. Data from the second and third waves were then analysed in sequence, following the previous coding structure. The longitudinal qualitative data analysis approach of Saldaña (2003) was used to examine the data. The "Framing questions" identified the changes that appeared from the previous wave. (e.g., What has changed and when and why it happened). The "descriptive questions" noted what increases or is cumulative across time and the 'surges' or 'epiphanies' that occurred. Forming and exploring the "Analytic/interpretive questions" later helped form the study's findings^21^. The study was reported according to the Consolidated Criteria for Reporting Qualitative Research (COREQ) checklist criteria for reporting qualitative research^24^.

### Ethical considerations

The study was approved by the Institutional Ethical Committee of the ICMR-Regional Medical Research Centre, Bhubaneswar (ICMR-RMRCB/IHEC-2020/027). All study participants were briefed on the study's objectives and invited for a telephonic or face-to-face interview at a time and place convenient for them, ensuring adherence to standard COVID-19 guidelines during the interview. It was emphasized that participation in the study is entirely voluntary, and all provided information would be treated confidentially. Informed consent was obtained before digitally recording the interview.The study adhered to the Declaration of Helsinki and later amendments. All research was performed in accordance with the ICMR National Ethical Guidlines for Biomedical and Health Research involving human partcipants.

## Results

The findings are presented under four major themes: (1) adapting clinical practice to ensure continuity of care, (2) appreciation of the multifaceted vulnerability of multimorbidity and need for attention, (3) adopting innovations for reaching out to patients, and (4) aligning with rapidly changing public health guidelines and health system interventions. The themes emerged by incorporating the Chronic Care Model and aligning it with the prevailing COVID-19 protocols during each wave, following the longitudinal qualitative data analysis approach as outlined by Saldaña. The coding tree—themes, categories, and codes that emerged across three waves of COVID-19 are provided in Table 4.

**Table 4:** Coding tree—themes, categories, and codes emerged across three waves of COVID-19.

Themes	First wave (March–November 2020)		Second wave (March–May 2021)		Third wave (January—March 2022)	
	Context	Responses	Context	Responses	Context	Challenges
Adapting clinical practice to ensure continuity of care	*COVID-19 was perceived as untreatable and life-threatening * RTPCR test started (Mar), but limited testing facilities *Patients could not be screened *No treatment guidelines for COVID-19 *No vaccination *Limited supply of protective equipment *Rapid antigen test & convalescent plasma therapy started (July), but not extensive	*Physicians feared getting infected and carrying the infection home *Every patient was a COVID-19 suspect, which brought fear *Extra precautions taken like wearing protective masks, face shields, and gloves *Continued work in unfavourable circumstances *Dissatisfied with the poor quality of patient consultations *Many experienced stress, disturbed sleep, anxiety, depression, and isolation *Chronic disease care was deprioritised *Most practices diverted for COVID-19 identification and care	*COVID-19 testing more available- RTPCR, Rapid antigen *COVID-19 screening could be done *Patients could be identified, and suspects isolated *COVID-19 is considered treatable for most, life-threatening for specific populations *Treatment guidelines for COVID-19 evolving *Most health workers getting vaccinated *Immunity against COVID-19 measured by antibody levels	*Fear of COVID-19 reduced after vaccination—but persisted for older physicians and those with multimorbidity *Patient care or chronic diseases resumed with precautions—Only extra care was needed for positive patients *COVID-19-positive screened to suggest emergency care and treatment at a COVID-19 facility or with home isolation	*COVID-19 testing is routinely done in suspected patients and before hospital admission *Effective treatment protocols for COVID-19 are followed *Most health workers and patients are vaccinated *Healthcare has opened up to deprioritise COVID-19 and consider other illnesses as well	*The COVID-19 virus is considered to have weakened *COVID-19 is compared to a Flu, with more severe symptoms *It is accepted as unavoidable in clinical practice *Physicians feel more protected by vaccinations
Appreciation of the multifaceted vulnerability of multimorbidity and need for attention	*Statewide lockdown and transport restrictions *Healthcare systems reorganised—Resources and health workers diverted to address COVID-19-related care	*Emergency care and specialised daycare services like dialysis, blood transfusion, radiotherapy, and chemotherapy continued amid strict COVID-19 related precautions *Non-COVID-19 illnesses, particularly chronic conditions, are not prioritised	*Vulnerability of patients with multimorbidity and chronic diseases to COVID-19 established *Chronic illnesses received renewed attention *Interdepartmental and other referrals became less *Holistic care could not be possible	*Emergency and essential chronic illness care continued *Physicians proactively looked for diabetes, hypertension, and other chronic illnesses *Additional COVID-19 precautions taken for them and encouraged to avoid hospital visits *When COVID-19 positive, those with multimorbidity were advised admission and heightened care *Primary care physicians, familiar with their patient's illnesses and could guide treatment	*The importance of addressing chronic illness and multimorbidity established	*Care for chronic illness continued as before pandemic *There was continued treatment priority and extra precaution for patients with a chronic illness or multimorbidity *These patients were prioritised for vaccination *There was an increase in hospital visits for these patients
Adopting innovations for reaching out to patients	*Routine consultations stopped *Lockdown, travel restrictions and precautionary measures reduced access to care	*Physicians could be approached remotely using mobile and internet networks *Primary physicians familiar with patient's illnesses guided patients in the absence of accessible medical records *This was considered a temporary solution for health emergencies	*Routine consultations are discouraged for chronic illness; remote consultations preferred *Consultations for chronic illness were limited to emergency and essential services such as dialysis and chemotherapy	*Physicians continued remote consultations could be reached out remotely through mobile phones, social media and WhatsApp *Hospitals started formal telemedicine facilities *Physicians guided home-based patient care and co-ordinated referrals *Specialist consultations in COVID-19 care settings used remote consultations *Remote consultations are considered an indispensable element in healthcare delivery *Having a patient database facilitated remote consultations *Remote consultations were extended to include counselling on home-based self-management of chronic illness	*Routine consultations resumed for chronic illness *Patients with chronic illness returned for formal consultations	*Physicians reduced the use of remote consultations *Hospitals-based formal telemedicine facilities continued to be used
Aligning with rapidly changing public health guidelines and health system interventions	*First case in Odisha *Pandemic declared a state disaster *Closure of places of gatherings in public *Creation of isolation facilities *Declaration of statewide lockdown and travel restrictions *Pandemic preparedness of health systems *Disrupted NCD programmes	*Initiation of COVID-19 diagnostic facilities *Expanded hospital and ICU facilities *Reorganised healthcare *Prioritised care for patients with chronic illness—e.g., cancer under chemotherapy/radiotherapy, renal failure under dialysis, those needing a transfusion *Promoted transport and online delivery of medicines for these patients *Promoted COVID-19 related awareness and advisory *Provided treatment support for home-based COVID-19 patients	*Lockdown and travel restrictions imposed from time to time *Chronic illness and multimorbidity identified as vulnerable to COVID-19	*Door-to-door surveillance of lung infection & comorbid chronic diseases Early diagnosis of COVID- 19 *COVID-19 vaccination was prioritised in older adults and those with multimorbidity *Rapid establishment of COVID-19 diagnostic labs across the state *Strengthening of healthcare facilities *Organising telemedicine consultations for all chronic diseases *Promoting the National digital health mission and emphasis on digital records	Lockdowns and travel restrictions less than before	*Extensive vaccination campaigns with a preference for older adults and those with multimorbidity *Widespread laboratory facilities set up for early COVID-19 identification and treatment * Health services were more intense than before NCD programmes resumed with a renewed focus

### Theme 1: adapting clinical practice to ensure continuity of care

#### Delivering care with fear and uncertainty

Physicians faced stressful conditions during the COVID-19 pandemic. In the early weeks of the pandemic, they reported feeling vulnerable, feared getting COVID-19, and taking the virus home to elderly family members with chronic illnesses. Many experienced symptoms of anxiety, depression, post-traumatic stress, disturbed sleep, isolation and even alienation from their community while working in the hospital. Nevertheless, most physicians continued work in public and private settings with precautions like wearing protective masks, face shields, and gloves or setting up a transparent barrier from the patient. They reported that these reduced the quality of patient examination and removed the personal touch. Most consultations included "seeing patients from a distance," where physicians could talk and see patients from far and prescribe. Consultation times were kept brief, exploring the essential aspects of the disease. Every incoming patient appeared as a carrier of the untreatable virus. The fear and uncertainty reduced over time as health care evolved to adjust to the pandemic.

“The treating physicians are going through much stress. ‘**Kichi loka hari galeni, kichi loka dari galeni**’ (some doctors have given up, others live in dread). Some have become so tired of maintaining these precautions that they do not want to continue working this way.” [P17, Nephrologist, June 2020]

#### Evolving patient care

With added efforts against COVID-19, the chronic disease services were sometimes overwhelmed. Primary care and general physicians adapted their practices well to the evolving pandemic. They were at the forefront of the efforts against COVID-19, updating themselves on the latest treatment protocols and adopting them quickly. They felt unquestioned liberty to use whatever available resources to treat their patients. The RTPCR (Real-Time Reverse Transcription—Polymerase Chain) test for COVID-19 was the mainstay for diagnosis, and physicians were more confident and less fearful when managing patients who tested negative. Patients suspected of having COVID-19 were isolated till the results came in. The introduction of Rapid antigen tests for COVID-19 and measuring serum antibodies further reduced feelings of vulnerability in physicians. Optimistic treatment guidelines and convalescent plasma therapy for COVID-19 aided their confidence. Nevertheless, all physicians acknowledged the advent of the COVID-19 vaccine as the turning point in the fight against the pandemic. Vaccinated physicians could see their patients without fear of getting infected. By the end of the third wave, COVID-19 was less discussed with dread.

“We are not afraid of COVID-19. It is a viral fever. It will not disappear; we must adapt and live with it. COVID-19 is now just a simple flu”. [P22, Internal Medicine physician, January 2022]

### Theme 2: appreciation of the multifaceted vulnerability of multimorbidity and need for attention

#### Shifting priorities in healthcare

During the pandemic, physicians revealed many shifts in the illness priorities among health administrators, health workers and patients. At the onset, healthcare systems were reorganised, and all resources were diverted for screening, isolating and treating COVID-19 patients. Health facilities created isolation areas and dedicated hospital beds for COVID-19. Other illnesses, particularly chronic conditions, were overlooked. During the statewide lockdown and transport restrictions, few patients with chronic illnesses visited hospitals. Physicians maintained treatment-continuity informal consultations through phones and WhatsApp. Doctors in specialities like nephrology and oncology reported that emergency care and specialised daycare services like dialysis, blood transfusion, radiotherapy, and chemotherapy continued amid strict COVID-19 related precautions. After the vulnerability of patients with chronic illness to COVID-19 was established, these patients received renewed attention. Physicians started looking proactively for diabetes, hypertension, and other chronic illnesses in all patients, ensuring heightened COVID-19-precautions and care for them. Patients with stable chronic illnesses were encouraged to avoid hospital visits. Hence, towards the later phases of the pandemic (August 2020 onwards), physicians noticed a surge in the return of patients who had not consulted for long periods.

“There is no disruption of service for dialysis or transplant follow-ups. Many who initially dropped out have somehow managed to come by private vehicles and ambulances.” [P9, Nephrology, June 2020]

#### Multimorbidity care—overcoming the complexities

As chronic illnesses received renewed attention, many physicians became aware that patients with multimorbidity were most vulnerable to COVID-19 and its complications. They took extra COVID-19 related precautions for such patients. Their routine consultations were deferred with a provisional treatment plan, prioritising the more severe ailments. Interdepartmental referrals became few, and a collaborative and coordinated approach to treatment planning and care was not always possible. Whenever a referral was not possible, physicians always consulted their colleagues or professional friends and started treatment for comorbid illnesses. Holistic care could not be possible. Some physicians, particularly primary care physicians, revealed that with a long therapeutic relationship, they were familiar with their patient's illnesses and could guide their treatment and referrals when illnesses worsened. The physicians ‘ familiarity with their patients complemented the limitations of remote consultations.

“Patients, like those with cancer, have many health issues, like developing arrhythmia, and we have to consult specialists over the phone about their management.” [P1, General Physician, April 2021]

### Theme 3: adopting innovations for reaching out to patients

#### Remote consultation

Most physicians praised the role of widespread mobile and internet connectivity in keeping them connected to their patients. They used mobile phones, social media, WhatsApp or teleconsultation platforms, with hospitals starting formal telemedicine facilities. There were exchanges of patient treatment information, investigation reports and prescriptions. Physicians sometimes referred patients to their nearest point of care for treatment, investigations, or medicines. Physicians and specialists also used similar remote consultation methods to manage COVID-19 patients in isolation, within the hospital or at home. Those centres managing chronic illness, having a database of treatment records, like dialysis and chemotherapy units, found the information helpful in contacting the patients and facilitating their remote consultations. At the pandemic's beginning (March 2020), remote consultations were considered a temporary solution for health emergencies. Consequently, it was hailed as an indispensable element in healthcare delivery. Nevertheless, as the COVID-19 pandemic waned and travel restrictions lifted, teleconsultation became less used.

“We must sensitise the patients regarding the availability of telemedicine as an option during normal times, and they can become more and more familiar with the facility.” [P4, Psychiatrist, April 2021]

#### Encouraging self-management

Most physicians encouraged self-management practices in patients by giving instructions on managing their chronic illnesses at home. They guided patients on medicines, diet, and exercise, and they taught patients and caregivers to identify symptoms of illnesses and carry out essential examinations like measuring blood pressure and blood glucose levels. They observed that illness self-management was not possible in frail elderly patients or those with dementia, depression, or other psychotic disorders, thereby increasing their care demand on family members.

“Many patients in isolation could follow instructions and manage their illnesses. Older patients and ones with psychiatric illnesses needed help.” [P7, Pulmonologist, April 2021]

### Theme 4: aligning with rapidly changing public health guidelines and health system interventions

The Government's initial response to the pandemic was to reduce viral transmission through lockdowns, prevention of gatherings, and restrictions on people's movement. Teleconsultation facilities were set up in all parts of the state, with consultants of various specialities. The rapid establishment of COVID-19 diagnostic laboratories also helped identify and separate COVID-19-infected patients from these vulnerable persons. Those COVID-19 patients with chronic illness were telephonically monitored by health workers and guided to the nearest COVID-19 hospital for better care. The pandemic initially disrupted NCD programs, affecting the active door-to-door campaigns to identify people with chronic illness, coordinate and deliver their treatment, and distribute medicines. With the rising importance of chronic illness care, there was a greater emphasis on these efforts and on keeping meticulous digital records of these patients. The Government encouraged the development of COVID-19 vaccines, first introduced among the older populations and those with a single chronic illness or multimorbidity. Eventually, COVID-19 vaccinations brought about a feeling of normalcy among patients with chronic illnesses and the physicians treating them.

“COVID-19 has helped us in one way and restricted us in another way. NCD training programs and screening stopped in the first wave, but later all these resumed. Health services were more intense than before.” [P5, Health administrator involved in the NCD program, January 2022]

## Discussion

The COVID-19 pandemic continued for an extended period, affecting chronic illness care. Our present study explored how physicians and health systems adapted to ensure continuity of care in patients with chronic illness and multimorbidity. During the first wave, physicians were hesitant to carry out routine consultations as they felt vulnerable and feared getting COVID-19. They conducted consultations by observing patients from a distance or with a barrier between them. Their familiarity with the patient compensated for the disadvantages of remote consultations. By providing instructions, they encouraged patients to manage their illnesses at home and on their own. Remote consultations were initially carried out informally over the phone. These were viewed as temporary solutions for medical emergencies and were aimed to provide continuity in care. Immediately after the first wave, teleconsultation facilities were set up across the state. The speedy establishment of COVID-19 diagnostic laboratories aided a gradual resumption of physical consultations. The RT-PCR test for COVID-19 was the gold standard for diagnosis, and doctors dealing with patients who tested negative felt more confident and less fearful of getting COVID-19 infection. The subsequent introduction of rapid antigen tests and serum antibody measurement further reduced physicians' sense of vulnerability. Vaccinated physicians could now treat patients without fear of getting infected with COVID-19. At the conclusion of the third wave, COVID-19 was less feared, and chronic disease care services were near normal.

Patients with chronic diseases and multimorbidity need care continuity to prevent complications, avoid emergencies, and maintain a better quality of life^7^. With a steady rise in the burden of chronic illnesses, the health systems in India are evolving to address these conditions through person-centred and collaborative care^8^. However, at the onset of the COVID-19 pandemic, physicians gave lesser priority to patients with chronic illnesses and multimorbidity. Nevertheless, their concerns increased when the vulnerability of these patients to COVID-19 became well-established^17,25,26^. This vulnerability is worsened by ageing and comorbid mental illness^27^. As evident from other studies, the emphasis on chronic illness care increased as the pandemic progressed, and this group of people were given heightened infection preventive measures at all levels^11,17^. These measures ranged from suggesting restricted social interactions, extra protection, and care in hospitals to being identified as the first group to receive the COVID-19 vaccine in India on the 16th of January 2021^28^. An emphasis on routine adult vaccination was also suggested for other infectious diseases^29,30^. The overhauled health system during the COVID-19 pandemic, like the added laboratory facilities and increased hospital and ICU beds, and the sensitised health providers provided a significant change in chronic illness and multimorbidity care and a relook at the government programs for NCDs.

Our study found physicians from diverse fields adapting and innovating to deliver patient care. However, the primary care physicians and general physicians stood out in the versatility of their role during the COVID-19 pandemic in addressing the health needs of patients with a chronic illness or multimorbidity. Their efforts were multifaceted and expanded beyond the scope of their routine work. These included identifying possible COVID-19 cases in these vulnerable groups, reaching out through remote consultations, educating patients about the virus and its prevention, providing mental health support, coordinating government interventions, and promoting and delivering vaccination. This immense adaptability and resilience while delivering care are also observed in other studies^31–33^. This crucial role of general physicians has also been noticed in the past during disasters like earthquakes, floods, cyclones, and other large-scale medical emergencies^34–37^. Though these medical emergencies and the current COVID-19 pandemic underline the importance of general physicians and family physicians as essential elements of a health system, their role is understated in medical education, and they are yet to develop as a discipline in India^38,39^ General physicians’ familiarity with the place of work, the people they treat and their therapeutic relationships have a crucial role to play in managing chronic illnesses during large-scale health emergencies.

Primary physicians with strong therapeutic relationships knew about the patient’s illness and provided support and guidance during remote consultations. These elements of care were crucial for holistic care in patients with multimorbidity by coordinating referrals to specialists and allied healthcare workers. As multimorbidity management calls for a realignment of healthcare through a framework of patient-centred medicine^40,41^, primary care physicians or family physicians form the crux of such restructuring. Patients can be down-referred from tertiary and secondary to primary care wherever possible^42,43^. Hence, primary care physicians are better positioned to coordinate health care delivery in chronic illnesses and multimorbidity. This study indicates that there is a need to foster collaborative efforts and effective communication among medical specialists and primary care physicians, as well as the sharing of treatment plans. Focusing on all aspects of a patient's multiple health conditions, this approach is essential for comprehensive and coordinated care.

During the pandemic, most physicians communicated with their patients through WhatsApp and mobile phones for remote consultations. These consultations provided an alternative to in-person clinical visits for chronic illness^44,45^. In these consultations, they encouraged patients to measure basic health parameters like blood pressure, temperature, oxygen saturation, and glucose levels to monitor treatment progress. Patients were taught to identify illness exacerbation and drug side effects besides informing on diet and exercises. Such remote consultations made patients more responsible for their illnesses. Such Patient activation and self-management practices found beneficial for chronic illnesses were encouraged during the COVID-19 pandemic^15,16,46,47^. These informal physician–patient remote consultations became more structured over time, and ethical and practice guidelines for teleconsultation were in place in the post-pandemic period.

Telemedicine evolved as a method of healthcare delivery for providing treatment instructions, improving patients' and caregivers' health literacy and self-care practices. The erstwhile Medical Council of India issued guidelines on telemedicine consultations in March 2020^48^. From the experiences of remote consultations during the COVID-19 Pandemic and instances of its use during other natural disasters, telemedicine can be an alternative mode of consultation during calamities when healthcare is disrupted^49^. Remote consultations for chronic diseases can also be done routinely to ensure care continuity and limit physical consultations to only those needing them, thereby ensuring health and reducing the burden on healthcare systems. The availability of electronic medical records can further the effectiveness of telemedicine in providing appropriate guidance for chronic illnesses and multimorbidity management. The Government of India introduced the Ayushman Bharat Digital Mission (ABDM), wherein a Health ID and Personal Health Records (PHR) can be created and linked with both public and private healthcare facilities^50^. With healthcare professionals across disciplines having better access to the patient's medical records, we can look forward to coordinated care for multiple chronic conditions.

Physicians play a pivotal role in guiding the self-management of illnesses, particularly among individuals with frailty or chronic conditions. This involves educating and sharing information with patients, developing personalized self-management plans, and encouraging patients to actively monitor their health. General Practitioners (GPs) are particularly vital in community healthcare, taking charge of direct care, decision-making, and referrals, and adapting their approaches during crises such as disasters. To fortify the healthcare system's resilience, it is crucial to invest in training programs for GPs and other healthcare professionals, focusing on enhancing comprehensive care and communication skills. The study underscores the importance of policymakers providing clearer guidance and support to clinicians during public health crises, especially when dealing with patients with multiple health issues. Standardized protocols, resource allocation strategies, and effective communication channels can facilitate decision-making. Additionally, both general physicians and specialists should be briefed on adapting their roles in crises. Addressing the increasing prevalence of multimorbidity requires a health delivery system that prioritizes patient-centred, holistic care, leveraging digital technology for telemedicine and shareable health records to enhance accessibility, particularly in underserved or remote areas.

In the context of public health crises, managing patients with multimorbidity requires a keen consideration of temporality—factors related to time such as disease progression, intervention timing, and changing patient needs. This becomes particularly complex when dealing with individuals having multiple concurrent health conditions. During events like pandemics, understanding the temporal dimension is crucial for prioritizing interventions, adapting healthcare resources, and addressing evolving patient needs. Clinicians play a pivotal role in navigating the temporal aspects of multimorbidity to optimize patient outcomes. The knowledge gained from managing temporality in one crisis can be invaluable for future public health emergencies, enabling healthcare systems to enhance preparedness and responsiveness. This learning process empowers clinicians to develop flexible and adaptive strategies, contributing to the creation of more resilient healthcare systems capable of effectively addressing unexpected challenges.

## Conclusions

The COVID-19 pandemic disrupted health systems affecting care for NCDs and multiple chronic conditions. However, physicians overcame their fears and found ways to deliver care amidst the chaos. Later, when chronic illnesses and multimorbidity emerged as conditions of vulnerability, there was a renewed focus on these conditions. To ensure care continuity, physicians leveraged their therapeutic relationships, used remote consultations and promoted disease self-management among patients. Health administrators and policymakers also initiated measures to protect persons with multiple chronic conditions, prioritising their healthcare and vaccination. This priority change and the added healthcare infrastructure and laboratory facilities may also boost chronic illness care. With the Government moving towards digital healthcare, teleconsultations can be essential during calamities and regular times for treating long-term conditions. The COVID-19 pandemic may be a watershed moment in the care for chronic conditions. The findings underscore the transformative potential of physician resilience and adaptation during the COVID-19 pandemic, emphasizing the importance of prioritizing patients with multimorbidity, incorporating teleconsultation, and implementing digital health records in healthcare systems to enhance chronic illness care and preparedness for future public health crises.

## Data Availability

All data generated or analysed during this study are included in this published article.
